# A Modern Medical Journal

**Published:** 1907-04-13

**Authors:** 


### The Hospital

A Journal of

The Medical Sciences and Hospital Administration.

New Series. No. 2, Vol. I. [No. 1074, Vol. XLII.] SATURDAY, APRIL 13, 1907.

### A Modern Medical Journal.

Would it fulfil any useful purpose to provide the medical profession with a modern medical journal? We believe it would, for the following reasons, amongst others. This is a busy age, which entails upon the individual worker the necessity to secure every facility which will economise time, whilst it supplies the necessary information and facilities for the acquirement of knowledge. The busy practitioner cannot devote a large amount of time to study, but he needs facilities to keep himself in touch with all that is going on in the medical world, and especially in the direction of the diagnosis and treatment of disease in all its aspects. The general practitioner has no doubt grievously felt the competition of the hospitals in recent years. Such competition would at once become relatively unimportant, if the practitioner who holds no hospital appointment could readily keep in touch with every new development. He would then be in a position to bring himself up to date by availing himself to the fullest extent of modern methods in all directions. The modern medical journal should be capable of supplying these wants of the practitioner upon a plan which should give him every facility for information, whilst it enables him to avail himself to the fullest extent of everything calculated to add to his knowledge.

It will be seen from an announcement which appears in to-day's Special Supplement, and which we hope everyone will read, that The Hospital, after nearly twenty-one years of successful work, proposes to make a new departure, which will constitute it the modern medical journal. In its new form The Hospital will include every section of hospital work. The object of the editors will be to supply an ably written, informative, readable, and practical medical journal, which will never contain more than thirty-two pages of literary matter, whilst it will be printed in long primer type, so as to form comfortable reading for the practitioner in his carriage, without the bulk and weight which are unavoidable in the case of a journal of from one hundred and fifty to two hundred pages. It may be felt that it may not be possible to cover all the ground or to include everything we desire to compass in the space to which we propose to limit ourselves. That it is possible, by the exercise of careful editing and clear writing to accomplish all this, will, we hope, be manifest by the contents of our present issue. The whole staff are thoroughly united and interested in the problem they have set themselves to solve. We have therefore full confidence, that, our readers will find, that the promise with which we have commenced this new series of The Hospital, will be more than fulfilled, before the year is out.

We are most anxious to secure the co-operation of the general body of the profession in this attempt to meet a want which we are assured they have long felt. It will be seen on reference to the last page of the present issue that we have specially set aside space for clinical communications from general practitioners, and that everybody who wishes to have an opening for the prompt publication of valuable monographs, based on clinical experience, will find it in the columns of The Hospital. Every contribution which is accepted for these sections will be paid for, the editors' wish being to make the new series of The Hospital of practical interest and value to every practitioner, whatever may be the conditions of his practice and surroundings. Finally it is hoped that the fact that there will be no increase in the price of The Hospital, and that it will be supplied to all subscribers, post free, for an inclusive payment of 13s. per annum in the United Kingdom, or of 17s. per annum in the colonies and abroad, will cause the enterprise thus displayed to appeal to the profession throughout the Empire. We cordially invite suggestions from every quarter, and pledge ourselves, in advance, to use every endeavour to meet the requirements of each branch of the profession to the fullest possible extent.

